# Metronomic Cyclophosphamide and Methotrexate Chemotherapy Combined with 1E10 Anti-Idiotype Vaccine in Metastatic Breast Cancer

**DOI:** 10.4061/2011/710292

**Published:** 2011-07-14

**Authors:** Jorge L. Soriano, Noyde Batista, Eduardo Santiesteban, Mayté Lima, Joaquín González, Robin García, Yohanka Zarza, María V. López, Myriam Rodríguez, Jorge L. Loys, Narciso Montejo, Frank Aguirre, Amparo Macías, Ana M. Vázquez

**Affiliations:** ^1^Oncology Service, Hermanos Amejeiras Hospital, Havana 10300, Cuba; ^2^Oncology Service, José R. López Tabrane Hospital, Matanzas 40100, Cuba; ^3^Clinical Division, Center of Molecular Immunology, Havana 11600, Cuba

## Abstract

The use of low doses of cytotoxic agents continuously for prolonged periods is an alternative for the treatment of patients with metastatic breast cancer who have developed resistance to conventional chemotherapy. The combination of metronomic chemotherapy with therapeutic vaccines might increase the efficacy of the treatment. Twenty one patients with metastatic breast cancer in progression and a Karnosky index ≥60%, were treated with metronomic chemotherapy (50 mg of cyclophospamide orally daily and 2.5 mg of methotrexate orally bi-daily), in combination with five bi-weekly subcutaneous injections of 1 mg of aluminum hydroxide-precipitated 1E10 anti-idiotype MAb (1E10-Alum), followed by reimmunizations every 28 days. Five patients achieved objective response, eight showed stable disease and eight had disease progression. Median time to progression was 9,8 months, while median overall survival time was 12,93 months. The median duration of the response (CR+PR+SD) was 18,43 months (12,20–24,10 months), being higher than 12 months in 76,9% of the patients. Overall toxicity was generally mild. Metronomic chemotherapy combined with 1E10-Alum vaccine immunotherapy might be a useful therapeutic option for the treatment of metastatic breast cancer due to its potential impact on survival and patient quality of live, low toxicity and advantages of the administration.

## 1. Introduction

Chemotherapeutic drugs given at the maximum tolerated dose (MTD) have had very low impact on the survival of patients with malignant metastatic diseases, with an increase of acute and chronic toxicities.

In the last ten years, several studies have demonstrated that chronic administration of low doses cytotoxic agents in more frequent schedules (metronomic delivery) was as effective as MTD regimens, but with reduced toxicity [1, 2]. 

Angiogenesis plays a central role in local tumor growth and in the development of distant metastases in breast cancer. Results of preclinical studies suggest that angiogenesis precedes the transformation process from hyperplasia to neoplasia [3]. 

Several chemotherapeutic agents, used routinely for breast cancer treatment, have antiangiogenic activity [4, 5]. While the objective of chemotherapeutic drugs administered at the MTD is to achieve optimal tumor cell killing, the highest antiangiogenic activity usually requires a prolonged exposition of low drug concentrations, as has been demonstrated in different preclinical models [6, 7]. The mechanism responsible of the anti-angiogenic activity of metronomic chemotherapy (MC) seems to be the induction of increased plasmatic levels of trombospondin 1, that is a potent endothelial angiogenic inhibitor [8].

MC can be considered as a variant of “dense-dose” treatment with the difference that cumulative dose of MC can be significantly lower than MTD-based chemotherapy [9]. MC reduces the level of toxicity and decreases or abolishes the need of support treatments with growth factors to accelerate recovery of bone marrow. In spite of having lower cumulative dose of drug, MC exhibits a superior therapeutic effect in terms of increase survival than conventional schemes of MTD [9]. 

In a previous published study [10], our group showed that MC with cyclophosphamide and methotrexate produced clinical responses and survival similar to those obtained with the schemes conventionally used in the treatment of breast cancer after a first line of chemotherapy for metastatic disease. A total of 28 patients were included in that study, and among them 14 patients demonstrated objective responses and 8 patients had disease stabilization. Median time to progression was 3,7 months (2,3–8,7 months), and median overall survival time was 7,9 months (95% CI, 3,7–26,2 months). Response duration was higher than 12 months in 18,2% of the patients. The most frequently observed toxicities were grade 2 anemia and leukopenia, which was observed in 11 patients; grade 2-3 liver toxicity in 6 patients; and grade 2-3 nausea in 9 patients.

On the other hand, the Center of Molecular Immunology (CIM) has reported a new vaccine preparation containing a murine anti-idiotype monoclonal (MAb) called 1E10 (racotumomab) obtained after immunization of BALB/c mice with P3 MAb, an antibody that recognizes N-glycolyl containing-gangliosides, sulphated glycolipids, and antigens present in melanoma, breast carcinomas, and lung tumours [11–14]. Preparations containing 1E10 MAb were able to induce antitumor effects in different tumor murine models [15].

A phase I clinical trial conducted in 15 patients with advanced breast cancer treated with 1 or 2 mg of 1E10-Alum vaccine every other week for six injections showed that there were no differences between the two levels of dose tested in relation to toxicity and immunogenicity. No evidence of serious or unexpected effects was observed. The median survival time of all treated patients was 19 months. For patients that received more than six doses of the anti-idiotype vaccine the median survival time was 24 months (95% confidence interval, 20.61–27.39 months). Two of the immunized patients remained with stable disease for more than 24 months [16]. 

More recently a preclinical study was carried out with the main objective to determine the antitumor effect of 1E10-Alum vaccine coadministered with low dose of cyclophosphamide in a murine mammary carcinoma model, based on their potentially shared antiangiogenic properties and/or a complementary proapoptotic effect by 1E10-Alum vaccine. The results of the study showed that the combination enhanced the efficacy of chemotherapy or immunotherapy alone in the F3II carcinoma model [17].

Here, we report the results of an exploratory study in metastatic breast cancer patients in which we evaluated the safety profile and the preliminary clinical outcome of the use of metronomic chemotherapy with cyclophosphamide and methotrexate in combination with 1E10-Alum vaccine.

## 2. Material and Methods

### 2.1. Patients

From February 2007 to February 2009, the Oncology Services of “Hermanos Ameijeiras” (HA) hospital (HAH), from Havana, Cuba and “José R. López Tabranes” (JRLT) hospital from Matanzas, Cuba carried out a prospective exploratory study that incorporated metronomic chemotherapy and 1E10-Alum vaccination to treat female patients with histologically confirmed metastatic breast cancer in progression, who had received at least one line of chemotherapy for their metastatic disease. Other eligibility criteria included Eastern Cooperative Oncology group (ECOG) performance ≤2 or Karnofsky index ≥60%, age ≥18 years and <80, normal haematopoietic, hepatic and renal functions, and life expectancy higher than 6 months, and adequate bone marrow reserve defined as leukocyte >3,000/*μ*l, neutrophiles >1,500/*μ*l (mm^3^); platelets >100,000/*μ*l (mm^3^); adequate renal function (serum creatinine >60 mL/min/1.73 m^2^) and hepatic function (total bilirubin and ALAT/ASAT ≤2.5 LSN). The most important exclusion criteria included the presence of brain metastases, pregnancy, or lactation, serious chronic diseases and active infections. Patients who had received treatment with MAbs or other biological modifiers of the immune response were not included.

Each patient included in this study gave a written informed consent. The study was approved by the Ethical Committees of the hospitals.

### 2.2. Anti-Idiotype 1E10 MAb for the Clinical Trial

1E10 MAb was purified from mouse ascites in the Good Manufacturing Practice (GMP) facilities of the Center of Molecular Immunology. Purification of 1E10 MAb was performed by DEAE-exchange chromatography followed by affinity chromatography on Protein A-CL Sepharose 4B column and size exclusion chromatography on Sephadex G-25 column (Amersham Pharmacia Biotech, Uppsala, Sweden). The purity of the isolated immunoglobulin was more than 97% as determined by SDS-PAGE, high-pressure liquid chromatography, and isoelectric focusing. The vaccine was produced in accordance with the Good Manufacturing Practice guidelines and certified by the Quality Control Department of the Center of Molecular Immunology. Briefly, sterile purified 1E10 MAb was aseptically mixed at a final concentration of 1 mg/mL with 5 mg/mL of aluminum hydroxide as adjuvant (Superfos Biosector, Frederikssund, Denmark). The mixture was gently stirred for 3 hours at room temperature. The aluminum hydroxide-precipitated MAb (1E10-Alum vaccine) was aliquoted into pyrogen-free, sterile glass vials and stored at 4°C until use. The final product was tested for sterility, pyrogenicity, and general safety in mice and guinea pigs before use, according to the United States Pharmacopoeia and to British Pharmacopoeia.

### 2.3. Treatment Schedule

Metronomic chemotherapy consisted in oral administration of cyclophosphamide at a dose of 50 mg once a day (after breakfast) and methotrexate at a dose of 2.5 mg twice a day on days 2 and 4 every week (08:00, 17:00 h). Antiemetic prophylaxis was performed with metoclopramide (10 mg/orally), thirty minutes before chemotherapy. 

1E10-Alum vaccine (1 mg/dose) was administered intradermally. Five doses were administered biweekly (2 months induction phase), followed by 10 monthly doses (maintenance phase).

Both treatments were maintained until patients had unacceptable toxicity or worsening of the performance status.

### 2.4. Evaluation before and during Treatment

Baseline evaluation included medical and smoking history, physical examination, vital signs, performance status, complete blood cell count and blood biochemistry, and plain chest radiographic examination, abdominal ultrasound, bone scan and selective radiographic examination and/or CT and/or MRI (depending on each case), breast ultrasound, and mammography (in case of need). Follow-up evaluation was done every 28 days before chemotherapy administration and included patients physical exam, complete blood count, and biochemistry. Imaging studies were performed according to patients symptoms, and if there were not any, every 3 months. 

The primary end point of this study was safety. Adverse events were evaluated according to the NCI Common Toxicity Criteria (version 3.0). In the evaluation of toxicity, only the greatest degree of toxicity was gathered and complications were recorded for each patient. 

The secondary end point of the study was the evaluation of preliminary efficacy in terms of response rate, time to progression, and overall survival. Response evaluation was performed according to Response Evaluation Criteria In Solid Tumors (RECIST, version 1.0), beginning at 4 weeks of starting treatment. Time to progression was calculated from the date of starting chemotherapy to date of the confirmation of progression or local or distant recurrence. Overall survival was the time between the beginning of chemotherapy and the date of death or the date at which patients were last known to be alive. Progression and survival times were estimated using the Kaplan-Meier method via the SPSS Program (version 10.0).

## 3. Results

Twenty-one patients were included in the study from February 2007 to February 2009. Median age was 46 years (range 35–82 years). More than 50% of the patients had a premenopausal status. All patients had measurable disease. The higher percentage of tumors was classified as estrogen receptor-positive and HER-2 receptor-negative. Less than 10% of the patients had a single metastatic site. The sites of metastases were, in decreasing order, bone, lung, skin, soft tissue, liver, and others (Table 1). The median number of previous chemotherapy lines received by the patients was 2 (range1–3). Nineteen patients had progressive metastatic disease, while two patients had stage IV disease at the moment of diagnosis. The fourteen patients included in the HA hospital had received at least two lines of conventional chemotherapy treatment for their metastatic disease, while the seven patients included by the JRLT hospital received only one line of chemotherapy. 

All patients were assessed for toxicity and the adverse events observed are shown in Table 2. In total, 40 adverse events were reported and most of them were classified as grade 1, according to the NCI Common Toxicity Criteria (version 3.0). Over 50% of side effects were pain at the site of injection. Four patients presented grade 2 toxicities: one patient had anemia, two patients had nausea and vomiting, and in one patient an increase in transaminase values was detected. Only one patient had to interrupt treatment due to grade 3 nausea and vomiting caused by the deterioration of general condition and progression of liver metastases.

Although this was an exploratory study, a preliminary evaluation of the clinical outcome was performed after patients were treated with the combination of MC and anti-idiotype vaccination (Table 3). Objective response (complete and partial responses) was demonstrated in 5 patients (23,8%), and the disease was stabilized in 8 patients (38,1%). Thus, the disease control rate was 61,9% (CR+PR+SD) after treatment. Regarding the patients with stage IV disease, one achieved complete remission, and then underwent local surgery of the breast (modified radical mastectomy). In the second patient the disease, was stabilized. 

The median time to disease progression was 9.82 months (CI 95%, 3,8–15,9 months), while the median survival time was 12.93 months (CI 95%, 7,8–19,9 months). The median duration of the response (CR+PR+SD) was 18,43 months (12,20–24,10 months), being higher than 12 months in 10 of the responding patients (76,9%).

## 4. Discussion

In the historical development of antineoplastic agents, it has been postulated that the cytotoxic effect on tumor cells of these agents is related to the dose administered and that this is the main cause of antitumor efficacy [18]. In fact, the principle of dose intensity is supported by experimental studies, where a small increment in drug dose can increase tumour cell death. This aspect of dose intensity and efficacy of tumour cell death in advanced breast cancer is still more controversial, due to the high heterogeneity of tumour breast cells, drug interactions, and the large number of prognostic factors that might impact in the outcome of treated breast cancer patients [19]. 

Some studies have demonstrated that cyclophosphamide when administered at MTD, causes endothelial cell apoptosis in the new formed tumor microvessels [20]. A detailed temporal analysis showed that endothelial cells were the first in the tumor to undergo apoptosis; however, this antiangiogenic effect does not translate into therapeutical benefit, because most of the damage in the tumor vasculature is repaired during the rest periods between the chemotherapy cycles [21, 22]. In contrast, MC which involves the frequent administration of low doses of cytotoxic drugs avoids dose-limiting adverse effects and then the need of rest periods, allowing the continue exposition of tumor endothelial cells to the actions of the cytotoxic drugs. For these reasons, chemotherapy in low doses has been evaluated in combination with other antineoplastic agents to minimize secundary effects and to produce synergic and additive benefits [23, 24].

In a recently published preclinical study [17], the treatment with a low-dose of cyclophosphamide had not effect in mice bearing a mammary tumor while a limited antitumor effect was induced by a single 1E10-Alum vaccine subcutaneous injection. However, the coadministration of the anti-idiotype vaccine with a low dose of cyclophosphamide significantly reduced the F3II mammary carcinoma growth. The therapeutic effect of the chemoimmunotherapy combination was associated to the increment of T cells infiltrating metastases, the reduction of new blood vessels formation and the increase of apoptotic tumor cells. These results support that the increase in apoptosis might contribute to tumor control [24] and that the antiangiogenic efficacy of a particular therapy might be increased with a combination of treatments [25–27].

In addition, it is known that metronomic regimen of cyclophosphamide does not only reduce tumor angiogenesis, but also selectively depletes immunosuppressive regulatory T cells allowing tumor progression control and facilitating antitumor immunotherapy [28]. The potential effect of the CT in the depletion of regulatory T cells could also contribute to increase the antitumor effects induced by 1E10-Alum vaccine in murine tumor models [17]. 

On the other hand, in a previous study 1E10-Alum treatment demonstrated to induce not only humoral, but also cellular immune responses in metastatic breast cancer patients [29]. Combined therapies that include this anti-idiotype vaccine might stimulate in cancer patients immunological mechanisms able to fight against tumor and its dissemination. 

This is the first report in the use of an anti-idiotype vaccine related to NeuGc-containing gangliosides in combination with MC for the treatment of metastatic breast cancer patients.

In the present study, although the patients were repeatedly treated with metronomic doses of cyclophophamide and methotrexate in combination with 1E10-Alum vaccine, only a low rate of side effects was observed, and these side effects were the same observed in previous reports using MC or anti-idiotype vaccine therapy in breast cancer patients [10, 16, 29].

The objective response rate was lower in this study than in the previous study conducted by our group using only MC [10] (14,3% versus 46,4%). This result could be related to the fact that, in comparison with the previous study, most of the patients included in the present study (66,6%) had received at least two therapeutic lines for metastatic disease and more than 90% had two or more metastatic sites. Nevertheless it is noteworthy that the median time to progression and the overall survival of the patients who entered this study and received MC and 1E10-Alum therapy were superior to the ones previously reported for the patients who received MC alone (9,82 versus 3,7 months and 12,93 versus 7,9 months, resp.).

Other encouraging result of the present study using the combined therapy of MC and the anti-idiotype vaccine was that the percentage of the patients who reached a response duration higher than 12 months was superior than the one reported when MC was used alone [10] (76,9% versus 18,2%).

In conclusion, the results of this study suggest that oral administration of low doses of cyclophosphamide and methotrexate, combined to immunotherapy with 1E10-Alum seems to prolong survival of a subgroup of patients with metastatic breast cancer with low toxicity. A randomized Phase II clinical trial will start soon where the clinical benefit of the combination of 1E10-Alum vaccine with MC in comparison with MC treatment alone will be evaluated.

## Figures and Tables

**Table 1:** Characteristics of patients included in the study.

Characteristic	No. of patients	Percent of total patients (%)
Median of age (range)	46 (35–82)	
Menopausal status (pre/post)	11/10	52,4/47,6
Estradiol Receptor		
Negative	5	23,8
Positive	9	42,9
Unknown	7	33,3
HER 2 Receptor		
Negative	13	61,9
Positive	1	4,8
Unknown	7	7
Patients by number of metastatic sites (1/2/>2)	2/11/8	9,5/52,4/38,1
Metastases localization (*n* = 50)		
(i) Lung/Pleura	10	47,6
(ii) Liver	6	28,6
(iii) Skin	7	33,3
(iv) Soft tissue	6	28,6
(v) Bone	12	57,1
(vi) Lymph nodes	5	23,8
(vii) Others	3	14,3

**Table 2:** Summary of the adverse events.

Event	Grade									
	0		1		2		3		4	
	No.	%	No.	%	No.	%	No.	%	No.	%
Leukopenia	19	90,5	2	9,5	0	0	0	0	0	0
Neutropenia	21	100	0	0	0	0	0	0	0	0
Anemia	18	85,7	2	9,5	1	4,8	0	0	0	0
Thrombocytopenia	21	100	0	0	0	0	0	0	0	0
Nausea/Vomiting	16	76,2	2	9,5	2	9,5	1	4,8	0	0
Transaminases	19	90,5	1	4,8	1	4,8	0	0	0	0
Gastric pain	19	90,5	2	9,5	0	0	0	0	0	0
Mucositis	21	100	0	0	0	0	0	0	0	0
Fever	19	90,5	2	9,5	0	0	0	0	0	0
Myalgia	18	85,7	3	14,3	0	0	0	0	0	0
Pain at the injection site	0	0	21	100	0	0	0	0	0	0

Total	191	82,7	35	15,2	4	1,7	1	0,4	0	0

**Table 3:** Efficacy evaluation after the treatment of MBC patients with MCT combined with 1E10 Aluminum/hydroxide vaccine.

Clinical response evaluation	MCT + 1E10 vaccine	
	*n* = 21	**%**
CR	2	9,5
PR	3	14,3
SD	8	38,1
PD	8	38,1
TTP (95% CI, months)	9,82 (3,8–15,9)	
OS (95% CI, months)	12,93 (7,8–19,9)	
Response duration >12 months	10	76,9%

## References

[1] Folkman J (1996). New perspectives in clinical oncology from angiogenesis research. *European Journal of Cancer Part A*.

[2] Emmenegger U, Man S, Shaked Y (2004). A comparative analysis of low-dose metronomic cyclophosphamide reveals absent or low-grade toxicity on tissues highly sensitive to the toxic effects of maximum tolerated dose regimens. *Cancer Research*.

[3] Schneider BP, Miller KD (2005). Angiogenesis of breast cancer. *Journal of Clinical Oncology*.

[4] Sweeney C, Sledge G (1999). Chemotherapy agents as antiangiogenic therapy. *Cancer Conference Highlights*.

[5] Slaton JW, Perrotte P, Inoue K, Dinney CPN, Fidler IJ (1999). Interferon-*α*-mediated down-regulation of angiogenesis-related genes and therapy of bladder cancer are dependent on optimization of biological dose and schedule. *Clinical Cancer Research*.

[6] Browder T, Butterfield CE, Kräling BM (2000). Antiangiogenic scheduling of chemotherapy improves efficacy against experimental drug-resistant cancer. *Cancer Research*.

[7] Hahnfeldt P, Folkman J, Hlatky L (2003). Minimizing long-term tumor burden: the logic for metronomic chemotherapeutic dosing and its antiangiogenic basis. *Journal of Theoretical Biology*.

[8] Hamano Y, Sugimoto H, Soubasakos MA (2004). Thrombospondin-1 associated with tumor microenvironment contributes to low-dose cyclophosphamide mediated endothelial cell apoptosis and tumor growth suppression. *Cancer Research*.

[9] Kerbel RS, Kamen BA (2004). The anti-angiogenic basis of metronomic chemotherapy. *Nature Reviews Cancer*.

[10] Soriano J, Lima M, González J (2009). Quimioterapia metronómica con ciclofosfamida y metotrexate en pacientes con cáncer de mama metastásico en progresión. *Revista Cubana de Medicina*.

[11] Vázquez AM, Pérez A, Hernández AM (1998). Syngeneic anti-idiotypic monoclonal antibodies to an anti-NeuGc-containing ganglioside monoclonal antibody. *Hybridoma*.

[12] Vazquez AM, Alfonso M, Lanne B (1995). Generation of a murine monoclonal antibody specific for N- glycolylneuraminic acid-containing gangliosides that also recognizes sulfated glycolipids. *Hybridoma*.

[13] Alfonso M, Díaz A, Hernández AM (2002). An anti-idiotype vaccine elicits a specific response to N-glycolyl sialic acid residues of glycoconjugates in melanoma patients. *Journal of Immunology*.

[14] Neninger E, Díaz RM, De La Torre A (2007). Active immunotherapy with 1E10 anti-idiotype vaccine in patients with small cell lung cancer: report of a phase I trial. *Cancer Biology and Therapy*.

[15] Vázquez AM, Gabri MR, Hernández AM (2000). Antitumor properties of an anti-idiotypic monoclonal antibody in relation to N-glycolyl-containing gangliosides. *Oncology Reports*.

[16] Díaz A, Alfonso M, Alonso R (2003). Immune responses in breast cancer patients immunized with an anti-idiotype antibody mimicking NeuGc-containing gangliosides. *Clinical Immunology*.

[17] Fuentes D, Avellanet J, Garcia A (2010). Combined therapeutic effect of a monoclonal anti-idiotype tumor vaccine against NeuGc-containing gangliosides with chemotherapy in a breast carcinoma model. *Breast Cancer Research and Treatment*.

[18] Kerbel RS, Yu J, Tran J (2001). Possible mechanisms of acquired resistance to anti-angiogenic drugs: implications for the use of combination therapy approaches. *Cancer and Metastasis Reviews*.

[19] Rahman ZU, Frye DK, Smith TL (1999). Results and long term follow-up for 1581 patients with metastatic breast carcinoma treated with standard dose doxorubicin-containing chemotherapy: a reference. *Cancer*.

[20] Orlando L, Cardillo A, Rocca A (2006). Prolonged clinical benefit with metronomic chemotherapy in patients with metastatic breast cancer. *Anti-Cancer Drugs*.

[21] Emmenegger U, Morton GC, Francia G (2006). Low-dose metronomic daily cyclophosphamide and weekly tirapazamine: a well-tolerated combination regimen with enhanced efficacy that exploits tumor hypoxia. *Cancer Research*.

[22] Johnston S, Stebbing J (2002). Breast cancer: metastatic. *Clinical evidence*.

[23] Hanahan D, Bergers G, Bergsland E (2000). Less is, more, regularly: metronomic dosing of cytotoxic drugs can target tumor angiogenesis in mice. *Journal of Clinical Investigation*.

[24] Lake RA, Robinson BWS (2005). Immunotherapy and chemotherapy—a practical partnership. *Nature Reviews Cancer*.

[25] Zhou H, Sequeira M, Goad MEP (2001). Efficacy and mechanisms of action of rmB7.2-Ig as an antitumor agent in combination with adriamycin and cytoxan chemotherapy. *Clinical Immunology*.

[26] Eralp Y, Wang X, Wang JP, Maughan MF, Polo JM, Lachman LB (2004). Doxorubicin and paclitaxel enhance the antitumor efficacy of vaccines directed against HER 2/neu in a murine mammary carcinoma model. *Breast Cancer Research*.

[27] Monzavi-Karbassi B, Pashov A, Jousheghany F, Artaud C, Kieber-Emmons T (2006). Evaluating strategies to enhance the anti-tumor immune response to a carbohydrate mimetic peptide vaccine. *International journal of molecular medicine.*.

[28] Ghiringhelli F, Menard C, Puig PE (2007). Metronomic cyclophosphamide regimen selectively depletes CD4^+^CD25^+^ regulatory T cells and restores T and NK effector functions in end stage cancer patients. *Cancer Immunology, Immunotherapy*.

[29] Guthmann MD, Castro MA, Cinat G (2006). Cellular and humoral immune response to N-glycolyl-GM3 elicited by prolonged immunotherapy with an anti-idiotypic vaccine in high-risk and metastatic breast cancer patients. *Journal of Immunotherapy*.

